# Advances in stereotactic navigation for pelvic surgery

**DOI:** 10.1007/s00464-017-5968-0

**Published:** 2017-12-06

**Authors:** A. R. Wijsmuller, L. G. C. Romagnolo, V. Agnus, C. Giraudeau, A. G. F. Melani, B. Dallemagne, J. Marescaux

**Affiliations:** 1 IRCAD/ EITS, Department of General, Digestive and Endocrine Surgery, Nouvel Hôpital Civil, University Hospital of Strasbourg, Strasbourg, France; 2 Department of Surgery, VU University Medical Center, Amsterdam, The Netherlands; 3 IRCAD Latin America, Department of Surgery, Barretos Cancer Center, Barretos, Brazil; 4 IHU Strasbourg, Institute of Image-Guided Surgery, Strasbourg, France; 5 Americas Medical City, Rio de Janeiro, Brazil

**Keywords:** Surgery, Computer-assisted, Stereotaxis techniques, Neuronavigation, Surgical procedures, Operative, Anatomy, Rectal neoplasms

## Abstract

**Background:**

Stereotactic navigation could improve the quality of surgery for rectal cancer. Critical challenges related to soft tissue stereotactic pelvic navigation include the potential difference in patient anatomy between intraoperative lithotomy and preoperative supine position for imaging. The objective of this study was to determine the difference in patient anatomy, sacral tilt, and skin fiducial position between these different patient positions and to investigate the feasibility and optimal set-up for stereotactic pelvic navigation.

**Methods:**

Four consecutive human anatomical specimens were submitted to repeated CT-scans in a supine and several degrees of lithotomy position. Patient anatomy, sacral tilt, and skin fiducial position were compared by means of an image computing platform. In two specimens, a 10-degree wedge was introduced to reduce the natural tilt of the sacrum during the shift from supine to lithotomy position. A simulation of laparoscopic and transanal surgical procedures was performed to assess the accuracy of the stereotactic navigation.

**Results:**

An up-to-supracentimetric change in patient anatomy was noted between different patient positions. This observation was minimized through the application of a wedge. When switching from supine to another position, sacral retroversion occurred independent of the use of a wedge. There was considerable skin fiducial motion between different positions. Accurate stereotactic navigation was obtained with the least registration error (1.9 mm) when the position of the anatomical specimen was registered in a supine position with straight legs, without pneumoperitoneum, using a conventional CT-scan with an identical specimen positioning.

**Conclusion:**

The change in patient anatomy is small during the sacral tilt induced by positional changes when using a 10-degree wedge, allowing for an accurate stereotactic surgical navigation. This opens up new promising opportunities to increase the quality of surgery for rectal cancer cases where it is difficult or impossible to identify and dissect along the anatomical planes.

**Electronic supplementary material:**

The online version of this article (10.1007/s00464-017-5968-0) contains supplementary material, which is available to authorized users.

## Introduction

Surgical navigation was developed by neurosurgeons who integrated medical imaging and stereotaxy [1]. It was reported to increase the safety and to minimize the invasiveness of surgical procedures by acting as a guidance tool using tracked surgical instruments in conjunction with preoperative images. It helps the surgeon to identify anatomical structures, which should be targeted or avoided. These systems are currently mainly used in brain, skull base, and vertebral surgery, and they have proven to be an essential adjunct to surgical procedures where anatomical landmarks are obscured and cannot be used for topographic orientation [2]. It could improve the quality of surgery for rectal cancer as shown when used in other contexts.

Recently, the performance of stereotactic navigation for minimally invasive transanal rectal surgery has been reported [3, 4]. Since anatomical structures at risk during rectal surgery are fixed retroperitoneally, they seem to be less affected by pneumoperitoneum and respiratory movements as compared to upper abdominal organs. However, pelvic surgery is associated with additional challenges as compared to surgical navigation in other context such as neurosurgery and orthopedic surgery. Rectal surgery is performed in patients with variable degrees of lithotomy, a position that is different from the supine position used for acquisition of preoperative imaging. This positional change could alter the patient anatomy and subsequently render stereotactic pelvic navigation using preoperative imaging inaccurate. Additionally, the motion of the skin reference points with their fiducial markers by means of positional change can hamper patient position registration in the operating room (OR) to begin with.

The objective of this study was to determine the difference in patient anatomy, sacral tilt, and fiducial marker position between these different patient positions and to investigate the feasibility and optimal set-up for stereotactic pelvic navigation.

## Materials and methods

### Imaging human anatomical specimens

**Fig. 1:**
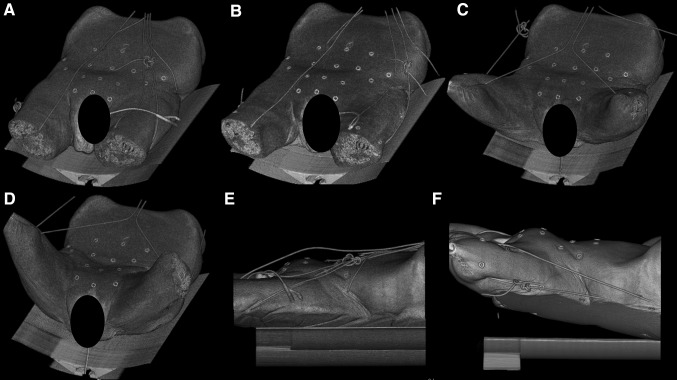
Different patient’s positions were investigated: supine, straight legs (**A**); Supine, hip abduction 60° (**B**); Hip flexion 45°, hip abduction 70° (**C**); Hip flexion 90°, hip abduction 80° (**D**); sagittal view without wedge (**E**); sagittal view with 10-degree wedge (**F**). Pneumoperitoneum as variable is not shown in this figure

Four consecutive experimental sessions were performed with four fresh-frozen human male anatomical specimens. These human anatomical specimens were submitted to repeated CT-scans at IHU (Institute of Image-Guided Surgery, University of Strasbourg, scanning technique is provided in supplementary text) to analyze the impact of various positions and pneumoperitoneum (Fig. 1; Table 1) on the change in patient anatomy, sacral tilt, and skin fiducials position. The upper and lower extremities of the specimens were removed with the proximal parts of the shafts of the humerus and femur still intact on each side. It allowed the acquisition of CT-scan images with the hip in several degrees of flexion, which is not realizable with conventional patients without limb amputations due to the width of the scanning tunnel. Hip flexion was generated by increasing/ decreasing the tension on a string fixed around the femoral shaft and the neck and/or around the shoulders of the specimen (Fig. 1). Hip abduction was generated with a wedge placed between the femoral shafts. The angle of hip flexion/ abduction was measured by means of a digital goniometer. Human anatomical specimen temperature was around room temperature at 20 °C.

**Table 1:** Four consecutive experimental sessions were performed with four human male anatomical specimens. For each specimen 4 different patient’s positions were investigated. For each position a CT-scan was obtained. In the first two specimens, pneumoperitoneum was a variable as well. The last two specimens were scanned with and without a 10-degree wedge

Experimental sessions 1 and 2		Experimental sessions 3 and 4^*^	
I	Supine, legs straight^†^, no pneumoperitoneum	I	Supine, legs straight^†^, no pneumoperitoneum
II	Supine, legs straight^†^, pneumoperitoneum	II	Supine, hip abduction 60°, no pneumoperitoneum
III	Hip flexion 90°, hip abduction 80°, pneumoperitoneum^‡^	III	Hip flexion 45°, hip abduction 70°, no pneumoperitoneum^‡^
IV	Hip flexion 90°, hip abduction 80°, no pneumoperitoneum^‡^	IV	Hip flexion 90°, hip abduction 80°, no pneumoperitoneum^‡^

*These scans were performed with or without a 10-degree wedge. A total of 4–8 scans were obtained per human anatomical specimen

†Hip adduction

‡Hip flexion: angle between femur axis and horizontal axis of the operation table

In stereotactic navigation, it is essential to obtain a perfect patient position registration in the OR by means of the navigation system. To do so, skin reference points are marked by means of radiopaque fiducials during preoperative CT-scanning, and these fiducials are left in place intraoperatively. Subsequently, after uploading these preoperative CT-scan images to the navigation system, the position of the patient can be registered via recognition of the fiducials using the optics of the navigation system. After this registration, the patient is tracked by a patient tracker which is fixed to the patient or to the operating table. Surgical instruments are tracked by an instrument tracker which is fixed to the instrument allowing the position of the tip of the instrument to be determined and visualized in the navigation scans. In these human anatomical specimens, a total of 12–18 skin reference points were marked with fiducials which were placed and fixed by means of sutures along the anterior bony landmarks of the pelvis and in between, assuming that skin motion at the level of the bony landmarks was the least. Bony landmarks included the anterior superior iliac spine and the pubic bone.

In the first two sessions, a 12 mmHg pneumoperitoneum induced through a 12 mm infra-umbilical trocar, was an additional variable. In the last two sessions, a forced sacral tilt was induced aiming at a reduction in the natural tilt of the sacrum during the shift from a supine to a lithotomy position, which was noted in the first two sessions. This was induced by a 10-degree wedge placed under the pelvis. The wedge was made from low-density foam, a material used to immobilize and reposition the body for radiation therapy.

### Image computing platform

**Fig. 2:**
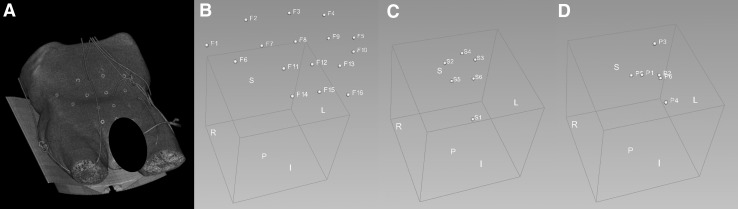
Markers were manually placed in the center of the skin fiducials in each CT-scan by using an image computing platform (3D Slicer) (**B**). The other markers S1-6 (**C**) and P1-6 (**D**) were placed at the anatomical landmarks as depicted in Table 2

**Fig. 3:**
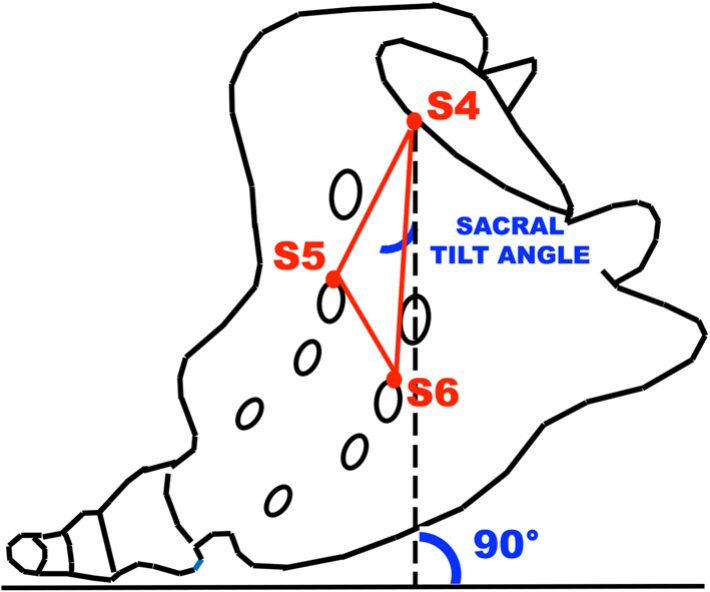
The sacral tilt angle is defined as the angle between the plane made up by S3–S5 and the vertical plane

To determine the change in patient anatomy, sacral tilt, and skin fiducial position, all CT data sets were edited. Predetermined anatomical landmarks (Table 2) and the center of each skin fiducial were marked by using an image computing platform (3D Slicer [5]) (Fig. 2, Video). The S1-6 points were chosen on the sacrum as a static structure allowing us to compare the sacral tilt. The P1-6 points correspond to pelvic organs of interest allowing us to compare their position in relation to the static point S4. To determine the reproducibility of marker placement, the same observer repeated a placement of S1–S6 markers in one CT data set three times. In the 2nd, 3rd, and 4th anatomical specimens urinary catheter was placed to facilitate the marking of specific landmarks. Skin fiducials were marked in all CT-scans of the last two sessions. The change in the following distances/ angles were measured through algorithms which were specifically designed for these outcomes and implemented in the 3D Slicer software:

- Patient anatomy: distances between the most centrally located bony S4 landmark and the six P1-6 points (Fig. 2D; Table 2),
- Sacral tilt angle: the angle of the plane between S3–S4–S5 and the vertical plane (Figs. 2C, 3),
- Skin fiducial position: distances between the most centrally located bony S4 landmark and the markers placed in the center of skin fiducials (Fig. 2B).

**Table 2:** Markers were manually placed in CT data sets on anatomical landmarks to analyze the change in patient anatomy and sacral tilt

Position pelvic organs		Sacral tilt	
P1	Most distal part ureter just before entering bladder, right side	S1	Distal tip sacrum
P2	Most distal part ureter, just before entering bladder, left side	S2	Ventral, upper edge foramen S1, right side
P3	Upper most cranial edge bladder	S3	Ventral, upper edge foramen S1, left side
P4	Lower most caudal edge prostate where urethra exists^*^	S4	Upper midline ventral rim sacral promontory
P5	Most cranial tip seminal vesicle, right side	S5	Ventral, upper edge foramen S2, right side
P6	Most cranial tip seminal vesicle, left side	S6	Ventral, upper edge foramen S2, left side

*in one anatomical specimen the most caudal edge of the prostate could not be identified. Therefore, in this case marker P4 was placed at an anatomical landmark consisting of a calcification in the prostate that could be identified in all scans

To test whether the mean sacral tilt angle changes between different positions, paired T-tests were performed to compare different positions. To test whether a 10-degree wedge influences the sacral tilt angle variation, a Fisher’s test was performed comparing the sacral tilt angle variation with and without the 10-degree wedge for different positions. To test whether a 10-degree wedge influences the patient anatomy, for each of the 6 distances (P1-6 to S4), the difference within variance was determined when using the 10-degree wedge as compared to the situation when no wedge was used.

### Laparoscopic and transanal stereotactic navigation

Alongside conventional CT-scans, a T2-weighted MRI scan and a C-arm CT-scan were also obtained during all tests for at least one position (the scanning technique is provided in a supplementary text). The MRI could be uploaded to the navigation system and fused with the scan used for registration in transanal navigation. At the final stage of each session, C-arm CT-scans were performed in a hybrid OR, and were used to register the position of the specimen alongside registration with conventional CT-scans in real-time. Since these C-arm CT-scans provide limited soft tissue resolution, conventional CT or MRI data sets were fused for transabdominal and transanal stereotactic navigation, respectively.

Each session was concluded with several simulations of laparoscopic and transanal surgery guided by means of stereotactic navigation (StealthStation^®^ S7 surgical navigation system, Medtronic Inc.; brain software). Three additional 5 mm trocars were introduced: 2 trocars in the right midclavicular line and 1 trocar in the left midclavicular line. Registration of the anatomical specimen position was performed with conventional CT data sets and with an intraoperative real-time C-arm CT-scan, in order to compare their accuracy. The patient tracker was fixed to the operating table adjacent to the pelvis or to the iliac bone at the level of the left anterior superior iliac spine in the first two and last two sessions, respectively. An MRI or conventional CT data set could be fused to navigate during a transanal or laparoscopic surgery in case of registration with C-arm CT-scan with low soft tissue resolution. Key anatomical landmarks were determined to test the navigation precision. The origin of the inferior mesenteric artery, the distal end of the aortic bifurcation and the tip of the promontory in laparoscopy and the exit point of the proximal urethra from the prostate in a transanal approach were identified surgically with an optical tracked instrument and correspondence on the navigation screen was evaluated.

## Results

A total of 24 conventional CT-scans were obtained, including 8 scans in the first two sessions and 16 scans in the last two experimental sessions of which 8 scans without and 8 scans with a 10-degree wedge. The global repositioning accuracy for the repeated placement of S1-6 markers in one scan three times was excellent with a mean displacement of 0.7 mm with a standard deviation 0.3 mm ensuring consistent placement.

### Patient anatomy

**Fig. 4:**
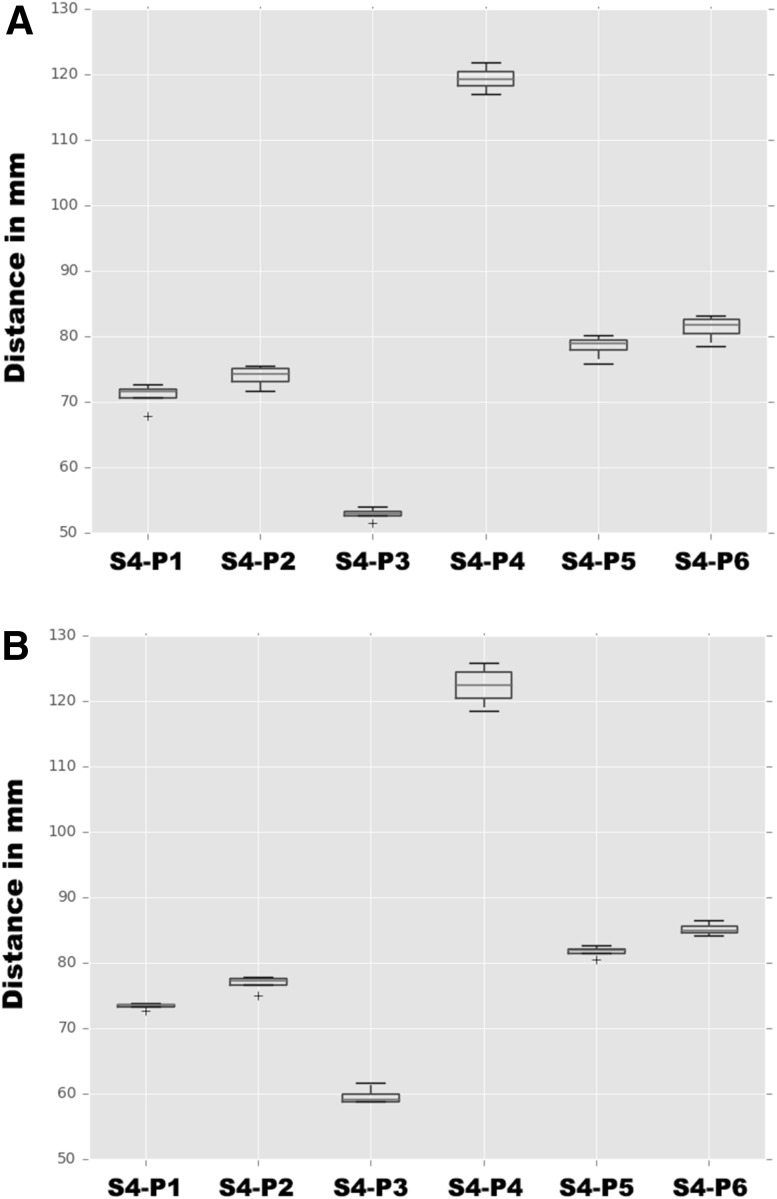
The distances between S4 and the six points, P1-P6, are depicted for the 3rd human anatomic specimen with (**A**) or without the wedge (**B**). The majority of the boxplots show a relative smaller width when using the 10-degree wedge as compared to when no wedge is used

An up-to-supracentimetric change in patient anatomy was noted between different positions for all human anatomical specimens. This observation was minimized through the application of a wedge (Fig. 4). For the third and fourth test, the change in patient anatomy was determined with and without a 10-degree wedge (Supplementary Table 1). For human anatomical specimen 3, Fisher’s test (with a 95% confidence) showed that there was a significant difference within variance for four distances (P2–S4, P3–S4, P5–S4, P6–S4) when using the 10-degree wedge as compared to when no wedge was used. Another Fisher’s test showed a significant difference for only one distance (P4–S4) for human anatomical specimen 4. The boxplots depicted in Fig. 4 illustrate this, having a relative smaller width when using the 10-degree wedge as compared to when no wedge was used.

### Sacral tilt

As compared to the supine position with straight legs, all other configurations result in an increase in the sacral tilt angle as depicted in Tables 3. With a 60-degree hip abduction, the sacrum already retroverts, up to more than 2 degrees. It also seems that pneumoperitoneum induction results in a decrease in sacral tilt. A paired T-test (with a 95% confidence) comparing the mean angle of sacral tilt in a supine position with straight legs with a 90-degree hip flexion position showed a significant difference. Another paired T-test comparing the mean angle of sacral tilt in a supine position with straight legs with a 60-degree hip abduction position, showed a significant difference. A Fisher’s test showed a significant difference when comparing the sacral tilt variation with and without a 10-degree wedge. For the third experimental session, the wedge resulted in more variations. In contrast, it resulted in fewer variations for the fourth session.

**Table 3:** The sacral tilt was determined between the plane formed by connecting the three points S4–S5–S6 and the vertical plane. The results of all the four human anatomical specimens are depicted

Human anatomical specimen	Sacral tilt angle (°)				Mean angle (sd)
	Supine, legs straight, no pneumoperitoneum	Supine, legs straight, pneumoperitoneum	Hip flexion 90°, hip abduction 80°, pneumoperitoneum	Hip flexion 90°, hip abduction 80°, no pneumoperitoneum	
1. Without wedge	27.6	29.1	28.8	28.4	28.5 (0.6)
2. Without wedge	20.8	21.2	21.4	21.2	21.2 (0.2)
	Supine, legs straight	Supine, hip abduction 60°	Hip flexion 45°, hip abduction 70°	Hip flexion 90°, hip abduction 80°	
3. Without wedge	5.9	6.5	6.5	6.0	6.2 (0.3)
3. With wedge	17.9	20.1	20.1	19.6	19.4 (0.9)
4. Without wedge	23.8	25.1	25.3	23.9	24.5 (0.7)
4. With wedge	37.3	38.2	38.5	38.6	38.2 (0.5)

### Skin fiducial position

As expected, displacement of skin fiducials was significantly impacted by pneumoperitoneum induction, rendering it useless in the perspective of optical navigation. This observation led to the abandonment of the method in the last two experiments. The distances between S4 and each individual skin fiducial were determined for the last two human anatomical specimens and are depicted in Supplementary tables 2–5. The standard deviation for these distances in the third session ranged from 0.4 to 2.9 mm. The standard deviation for these distances in the fourth session ranged from 0.7 to 6.1 mm.

### Stereotactic navigation

The results from stereotactic navigation are attached in the Supplementary Table 6. The smallest registration errors, both of 1.9 mm, were recorded in case of two registrations of the positions without pneumoperitoneum in supine position with straight legs by means of a conventional CT-scan with an identical specimen positioning.

## Discussion

This is the first study to evaluate the impact of a changing patient position as required by rectal surgery on the patient anatomy, sacral tilt, and skin fiducials position. The study provides essential anatomical and dynamic data, which may open the way for the use of current optical navigation systems in pelvic organ surgery. These data can serve as a basis for an optimal registration process in order to prevent inaccurate registration, which can render a guidance system worse than useless and even dangerous [6].

We introduce a new method to determine the change in patient anatomy, sacral tilt, and skin fiducial position using an image computing platform, hence facilitating computerized quantitative image analysis. Markers were placed manually by using a 3D Slicer software. An excellent repositioning accuracy for the repeated placement of S1-6 markers in one scan three times suggests a proper method for marker placement.

Other studies which have contributed to the knowledge of pelvic organ motion mainly include studies in radiation therapy. Multiple studies have been performed to quantify the motion of pelvic organs such as prostate, bladder, and vagina in order to optimize the planning of the volume which should be targeted by radiation to compensate for tissue deformation [7, 8]. The role of rectal distension on prostate motion had also been investigated [9].

Studies looking into prostate position demonstrated motion of the prostate as a result of motion at the level of the legs. One study investigating prostate motion reported that immobilization at the level of the patient’s legs in a supine position improves the set-up accuracy of radiotherapy as compared to an immobilization at pelvic level [7]. Other factors influencing pelvic organ motion include rectal distension. Strong evidence has shown that rectal distension on treatment planning CT-scan decreased the probability of biochemical and local control in prostate cancer patients who were treated with radiotherapy without daily image-guided prostate localization [9].

Most of these studies compared 3D organ contours between different scans [8]. This technique could be accurate provided that there is no change in organ shape and provided that there are no geometrical uncertainties. However, positional change is the subject of our investigation and consequently, we cannot assume that there is no change. Additionally, segmentation of organ contours is more time-consuming and difficult as compared to marking certain landmarks.

The experimental protocol was tailored on the results of the first two experimental sessions. The pneumoperitoneum was abandoned as a variable in the last two human anatomical specimens since it was shown to be associated with extensive displacement of skin fiducials. The 10-degree wedge was introduced since a sacral retroversion was noted when hip flexion and/or abduction was applied in the first two sessions. Our hypothesis that the wedge decreases sacral tilt was not confirmed since a sacral retroversion was also noted with wedge use. However, interestingly, pelvic organ motion was reduced significantly, suggesting a benefit from using a wedge.

Based on our experiments, we can conclude that the following aspects should be taken into account and included in the protocol for an optimal set-up of point-merge stereotactic navigation in pelvic surgery: patient position registration should be performed without pneumoperitoneum in a similar patient position to the position during preoperative CT-scanning with fiducials. This is because a changing patient position results in skin fiducial motion hampering accurate patient position registration. A supine position with straight legs is the preferred position. The patient tracker should be fixed into the iliac bone to integrate the change in sacral tilt angle into the surgical navigation system since a change is expected to occur when switching position. Finally, a forced sacral tilt seems to minimize the change in patient anatomy.

Limitations related to stereotactic navigation include the need for maintaining a direct line of sight between the infrared camera of the navigation system and the patient and instrument tracker. This line of sight can be hampered by the legs of the patients that are placed in lithotomy and the surgeon that is positioned between the patient’s legs. Another limitation is that stereotactic navigation relies on preoperative images for accurate navigation. Therefore, real-time geometric changes in pelvic anatomy caused by for example tissue dissection and traction are known to affect the accuracy of stereotactic navigation.

Other factors which should be considered based on earlier studies on pelvic organ motion are the following: rectal and bladder volume should be equal during the scans which are used for registration/navigation, as well as intraoperatively. Consequently, the bladder should be emptied before scanning as well as intraoperatively via the placement of a catheter. The rectum should be emptied by means of an enema. In case of transanal TME, the rectum should be emptied just before closing the pure-string. The pelvic diaphragm muscle tension should be equal during the scans, as well as intraoperatively.

Future directions which can further improve the accuracy of stereotactic pelvic navigation include the use of registration methods which do not require skin fiducials and which use real-time intraoperative imaging to perform patient position registration. A patient tracker which can be fixed to the pelvis and scanned intraoperatively would eliminate the need for skin fiducials along with its associated risks of imprecision. Nowadays, intraoperative CT-scan guidance is facilitated with the use of hybrid ORs which are equipped with real-time C-arm CT-scans. However, the diameter of the current real-time C-arms CT-scan is a limiting factor since it is too small to scan a patient positioned in lithotomy. Other imaging techniques which have been reported and which are suggested to improve accuracy providing a continuous intraoperative real-time feedback include US imaging which can be fused with CT or MRI data sets [10].

Surgical navigation is likely to improve the accuracy and efficiency of rectal surgical procedures in which it is difficult or impossible to identify and dissect along the anatomical planes. In conclusion, the current study shows that accurate stereotactic surgical pelvic navigation is feasible when taking into account several aspects of registration set-up.

## Electronic supplementary material

Below is the link to the electronic supplementary material.

Supplementary material 1 (DOCX 32 KB)

Supplementary material 2 (M4V 44933 KB)
