# Supplementary material for: Advances in stereotactic navigation for pelvic surgery
Source: Surg Endosc. 2017 Dec 6;32(6):2713–20. doi: 10.1007/s00464-017-5968-0 (PMC5956093; doi:10.1007/s00464-017-5968-0)
Supplement: Supplementary file 1 — Supplementary material 1 (DOCX 32 KB) [file 464_2017_5968_MOESM1_ESM.docx]

**Supplementary material**

**Supplementary Text**

Conventional CT, C-arm CT and MRI technique:

- Conventional CT technique : The CT examination of abdomen and pelvis were performed on Somatom definition AS 128 slice (Siemens, Forchheim, Germany). The following technical parameters were used for the examination : 140 kV and 205 mAs effective, slice width of 1mm, a pitch 0.8 mm, FOV 512*512.

- C-arm CT technique : Artis Zeego, X-ray tube MEGALIC Cat Plus. Flat detector as40 with active imaging size of 382mm*296mm. Different parameters for each 3D rotational acquisition. One example would be : 71 kV, 391 mA, 6 seconds duratuion of acquisition, 60 images/seconds, FOV 512*512.

- MRI technique : The specimens were also examined with a 1.5 Tesla MR system (Aera, Siemens Healthcare, Erlangen, Germany) using a 18-channel phased-array flexible coil and a spine coil for signal reception. A 3D T2-weighted SPACE (Sampling Perfection with Application of optimized Contrasts using different flip-angle Evolutions) sequence was acquired in all specimens with the following parameters: field of view (FOV) 375*305mm², echo time (TE) 145ms, repetition time (TR) 2000ms, acceleration factor GRAPPA 2, in plane resolution interpolated to 0.73*0.73mm², 208 1mm-thick axial slices. In order to counteract the weak signal and contrast yielded by dead tissues, 3.4 excitations were used, leading to an acquisition time (TA) of 18min30. Additionally, T1 images were acquired on the 2 first specimens with the same parameters, except TE/TR=15/400ms, leading to TA=11min.

| Distances | Human anatomical specimen 3 | | | | Human anatomical specimen 4 | | | |
| --- | --- | --- | --- | --- | --- | --- | --- | --- |
|  | Without wedge | | With wedge | | Without wedge | | With wedge | |
|  | Mean (mm) | Sd | Mean (mm) | Sd | Mean (mm) | Sd | Mean (mm) | Sd |
| P1-S4 | 71.0 | 1.8 | 73.4 | 0.4 | 59.7 | 0.8 | 59.8 | 0.5 |
| P2-S4 | 74.0 | 1.5 | 76.9 | 1.1 | 60.8 | 1.6 | 60.5 | 0.9 |
| P3-S4 | 52.9 | 0.9 | 59.7 | 1.1 | 36.9 | 1.9 | 38.7 | 1.2 |
| P4-S4 | 119.4 | 1.8 | 122.3 | 2.8 | 120.5 | 1.2 | 116.4 | 1.2 |
| P5-S4 | 78.5 | 1.6 | 81.8 | 0.8 | 55.9 | 1.5 | 55.3 | 0.4 |
| P6-S4 | 81.3 | 1.8 | 85.2 | 0.9 | 58.4 | 1.1 | 56.9 | 0.4 |

Supplementary Table 1

Distances in millimetres from P1-P6 to point S4, for the third and fourth test, with or without a wedge. The standard deviation decreases when using a wedge.

| Fiducials | Distance fiducial to point S4 (mm) | | | | Mean distance in mm (sd) |
| --- | --- | --- | --- | --- | --- |
|  | Position 1 | Position 2 | Position 3 | Position 4 |  |
| Fiducial 1 | 86.0 | 85.5 | 85.6 | 84.8 | 85.5 (0.4) |
| Fiducial 2 | 70.8 | 66.2 | 67.5 | 67.0 | 67.9 (1.8) |
| Fiducial 3 | 92.9 | 92.1 | 92.4 | 92.9 | 92.6 (0.4) |
| Fiducial 4 | 141.2 | 141.1 | 142.2 | 141.9 | 141.6 (0.5) |
| Fiducial 5 | 122.4 | 123.9 | 121.7 | 120.7 | 122.2 (1.2) |
| Fiducial 6 | 110.7 | 112.4 | 110.1 | 109.1 | 110.6 (1.2) |
| Fiducial 7 | 116.9 | 114.6 | 115.1 | 111.9 | 114.6 (1.8) |
| Fiducial 8 | 123.0 | 119.7 | 121.8 | 117.8 | 120.6 (2.0) |
| Fiducial 9 | 114.5 | 113.1 | 113. 0 | 108.6 | 112.3 (2.2) |
| Fiducial 10 | 107.1 | 106.2 | 106.2 | 103.3 | 105.7 (1.4) |
| Fiducial 11 | 118.4 | 117.9 | 117.6 | 116.2 | 117.5 (0.8) |
| Fiducial 12 | 140.7 | 138.8 | 140.0 | 140.5 | 140.0 (0.7) |
| Fiducial 13 | 133.4 | 132.9 | 133.9 | 133.2 | 133.3 (0.4) |
| Fiducial 14 | 134.3 | 132.5 | 132.3 | 129.5 | 132.2 (1.7) |
| Fiducial 15 | 133.4 | 132.7 | 134.4 | 128.7 | 132.3 (2.2) |
| Fiducial 16 | 116.1 | 114.0 | 115.7 | 117.9 | 115.9 (1.4) |
| Fiducial 17 | 104.0 | 101.1 | 102.2 | 103.6 | 102.7 (1.2) |
| Fiducial 18 | 108.2 | 105.5 | 107.8 | 108.2 | 107.4 (1.1) |

Supplementary Table 2

Distances in millimetres from skin fiducial 1-18 to point S4, third test, with a wedge.

| Distances | Distance fiducial to point S4 (mm) | | | | Mean distance in mm (sd) |
| --- | --- | --- | --- | --- | --- |
|  | Position 1 | Position 2 | Position 3 | Position 4 |  |
| Fiducial 1 | 90.3 | 89.8 | 88.3 | 88.8 | 89.3 (0.8) |
| Fiducial 2 | 71.7 | 70.9 | 69.9 | 70.1 | 70.7 (0.7) |
| Fiducial 3 | 90.3 | 91.5 | 90.1 | 88.7 | 90.1 (1.0) |
| Fiducial 4 | 143.5 | 142.2 | 142.2 | 143.1 | 142.7 (0.6) |
| Fiducial 5 | 124.1 | 123.4 | 122.9 | 121.8 | 123.1 (0.8) |
| Fiducial 6 | 112.2 | 112.1 | 111.1 | 108.7 | 111.0 (1.4) |
| Fiducial 7 | 116.0 | 113.3 | 113.6 | 109.4 | 113.1 (2.4) |
| Fiducial 8 | 121.7 | 119.6 | 120.1 | 116.4 | 119.4 (1.9) |
| Fiducial 9 | 112.3 | 111.0 | 110.7 | 104.8 | 109.7 (2.9) |
| Fiducial 10 | 103.7 | 104.2 | 103.4 | 100.7 | 103.0 (1.4) |
| Fiducial 11 | 115.1 | 116.5 | 115.0 | 114.0 | 115.1 (0.9) |
| Fiducial 12 | 137.1 | 138.0 | 136.9 | 136.6 | 137.1 (0.5) |
| Fiducial 13 | 133.2 | 131.0 | 134.1 | 131.1 | 132.4 (1.3) |
| Fiducial 14 | 133.6 | 130.9 | 131.0 | 128.7 | 131.0 (1.7) |
| Fiducial 15 | 133.0 | 132.6 | 133.1 | 126.2 | 131.2 (2.9) |
| Fiducial 16 | 122.9 | 123.1 | 121.0 | 123.2 | 122.5 (0.9) |
| Fiducial 17 | 111.0 | 111.1 | 108.3 | 110.0 | 110.1 (1.1) |
| Fiducial 18 | 109.1 | 109.9 | 108.4 | 108.5 | 109.0 (0.6) |

Supplementary Table 3

Distances in millimetres from skin fiducial 1-18 to point S4, third test, without a wedge.

| Distances | Distance fiducial to point S4 (mm) | | | | Mean distance in mm (sd) |
| --- | --- | --- | --- | --- | --- |
|  | Position 1 | Position 2 | Position 3 | Position 4 |  |
| Fiducial 1 | 85.4 | 85.9 | 87.1 | 88.6 | 86.8 (1.3) |
| Fiducial 2 | 79.1 | 79.5 | 79.0 | 81.5 | 79.8 (1.0) |
| Fiducial 3 | 111.7 | 110.9 | 107.4 | 109.7 | 109.9 (1.6) |
| Fiducial 4 | 138.7 | 140.2 | 141.5 | 141.5 | 140.5 (1.2) |
| Fiducial 5 | 128.9 | 131.9 | 130.8 | 130.2 | 130.4 (1.1) |
| Fiducial 6 | 123.8 | 123.6 | 118.3 | 113.5 | 119.8 (4.3) |
| Fiducial 7 | 126.3 | 126.5 | 119.7 | 113.4 | 121.4 (5.4) |
| Fiducial 8 | 140.8 | 139.6 | 132.2 | 128.0 | 135.2 (5.3) |
| Fiducial 9 | 142.0 | 141.0 | 137.1 | 136.3 | 139.1 (2.4) |
| Fiducial 10 | 154.1 | 154.9 | 150.4 | 150.9 | 152.6 (1.9) |
| Fiducial 11 | 156.5 | 155.5 | 151.1 | 144.4 | 151.9 (4.8) |
| Fiducial 12 | 153.7 | 153.5 | 147.8 | 142.9 | 149.5 (4.5) |
| Fiducial 13 | 166.9 | 165.3 | 160.3 | 151.5 | 161.0 (6.0) |
| Fiducial 14 | 101.5 | 100.9 | 104.6 | 107.0 | 103.5 (2.5) |
| Fiducial 15 | 96.8 | 97.4 | 95.7 | 100.5 | 97.6 (1.8) |
| Fiducial 16 | 127.9 | 128.5 | 126.1 | 128.8 | 127.8 (1.1) |

Supplementary Table 4

Distances in millimetres from skin fiducial 1-18 to point S4, fourth test, with a wedge.

| Distances | Distance fiducial to point S4 (mm) | | | | Mean distance in mm (sd) |
| --- | --- | --- | --- | --- | --- |
|  | Position 1 | Position 2 | Position 3 | Position 4 |  |
| Fiducial 1 | 85.6 | 87.0 | 86.6 | 85.2 | 86.1 (0.7) |
| Fiducial 2 | 78.1 | 79.7 | 80.6 | 78.3 | 79.2 (1.0) |
| Fiducial 3 | 111.6 | 112.1 | 112.6 | 109.5 | 111.5 (1.2) |
| Fiducial 4 | 135.5 | 138.1 | 139.1 | 138.6 | 137.8 (1.4) |
| Fiducial 5 | 128.7 | 131.0 | 132.5 | 127.5 | 129.9 (1.9) |
| Fiducial 6 | 125.6 | 126.8 | 126.8 | 119.9 | 124.8 (2.9) |
| Fiducial 7 | 128.1 | 130.3 | 129.3 | 121.4 | 127.3 (3.5) |
| Fiducial 8 | 142.4 | 142.8 | 142.1 | 133.4 | 140.2 (3.9) |
| Fiducial 9 | 143.8 | 144.2 | 143.2 | 138.2 | 142.2 (2.4) |
| Fiducial 10 | 154.0 | 154.1 | 154.8 | 150.5 | 153.4 (1.7) |
| Fiducial 11 | 161.7 | 162.5 | 157.6 | 149.1 | 157.7 (5.3) |
| Fiducial 12 | 156.9 | 157.9 | 154.5 | 149.0 | 154.6 (3.4) |
| Fiducial 13 | 172.7 | 171.4 | 168.0 | 157.3 | 167.4 (6.1) |
| Fiducial 14 | 99.4 | 98.5 | 99.3 | 100.9 | 99.5 (0.9) |
| Fiducial 15 | 96.0 | 92.4 | 93.4 | 92.9 | 93.7 (1.4) |
| Fiducial 16 | 124.5 | 126.3 | 126.6 | 123.9 | 125.3 (1.1) |

Supplementary Table 5

Distances in millimetres from skin fiducial 1-18 to point S4, fourth test, without a wedge.

| Experimental session | Position in OR | Registration scan | Position during registration scan | Merged scan if applicable | Registration error (mm) | Accurate navigation |
| --- | --- | --- | --- | --- | --- | --- |
| 1 | Hip flexion 90°, hip abduction 80°, pneumoperitoneum | Conventional CT | Hip flexion 90°, hip abduction 80°, pneumoperitoneum | NP | 3.6 | NP |
|  | Hip flexion 90°, hip abduction 80°, pneumoperitoneum | Conventional CT | Supine, legs straight, no pneumoperitoneum | NP | 4.0 | No* |
|  | Hip flexion 90°, hip abduction 80°, pneumoperitoneum | C-arm CT | Hip flexion 90°, hip abduction 80°, pneumoperitoneum | MRI, hip flexion 90°, hip abduction 80°, pneumoperitoneum | 2.4 | Yes |
| 2 | Hip flexion 90°, hip abduction 80°, pneumoperitoneum | C-arm CT | Hip flexion 90°, hip abduction 80°, no pneumoperitoneum | NP | 2.4 | NP |
|  | Hip flexion 90°, hip abduction 80°, pneumoperitoneum | Conventional CT | Supine, legs straight, no pneumoperitoneum | MRI, supine, legs straight, no pneumoperitoneum | 3.8 | Yes |
| 3 | Supine, legs straight, no pneumoperitoneum | Conventional CT | 3 | NP | 1.9 | Yes^†^ |
|  | Hip flexion 90°, hip abduction 80°, no pneumoperitoneum | Conventional CT | Hip flexion 90°, hip abduction 80°, no pneumoperitoneum | NP | 2.4 | Yes^†^ |
|  | Wedge 10°, supine, legs straight, no pneumoperitoneum | Conventional CT | Wedge 10°, supine, legs straight, no pneumoperitoneum | NR | 2.4 | NR |
| 4 | Supine, legs straight, no pneumoperitoneum | Conventional CT | Supine, legs straight, no pneumoperitoneum | NP | 1.9 | Yes |
|  | Hip flexion 45°, hip abduction 70°, no pneumoperitoneum | Conventional CT | Hip flexion 45°, hip abduction 70°, no pneumoperitoneum | NP | 2.4 | Yes |
|  | Wedge 10°, supine, legs straight, no pneumoperitoneum | C-arm CT | Wedge 10°, supine, legs straight, no pneumoperitoneum | CT, wedge 10°, supine, legs straight, no pneumoperitoneum | NR | Yes |
|  | Wedge 10°, supine, legs straight, no pneumoperitoneum | Conventional CT | Wedge 10°, supine, legs straight, no pneumoperitoneum | NP | 2.1 | Yes |
|  | Wedge 10°, hip flexion 45°, hip abduction 70°, no pneumoperitoneum | Conventional CT | Wedge 10°, hip flexion 45°, hip abduction 70°, no pneumoperitoneum | NP | 2.7 | Yes |
|  | Hip flexion 45°, hip abduction 70°, no pneumoperitoneum | Conventional CT | Hip flexion 45°, hip abduction 70°, no pneumoperitoneum | NP | 2.7 | Yes |

* a two cm shift was noted between the instruments on the navigation screen and the aortic bifurcation

† Because of abdominal aortic aneurysm no origin of the AMI could be identified. The aortic bifurcation, common iliac artery, iliac bifurcation, the level of the promontory and the proximal part of the urethra were identified

NR= not reported

NP= not performed

Supplementary Table 6

Setups for stereotactic navigation. Registration of the position of the specimen was performed through point merge registration in all cases. Registration errors were recorded and an attempt at navigation was done and whether this was successful was noted.
